# Supplementary figures and images for: Is YouTube promoting the exotic pet trade? Analysis of the global public perception of popular YouTube videos featuring threatened exotic animals
Source: PLoS One. 2021 Apr 13;16(4):e0235451. doi: 10.1371/journal.pone.0235451 (PMC8043400; doi:10.1371/journal.pone.0235451)

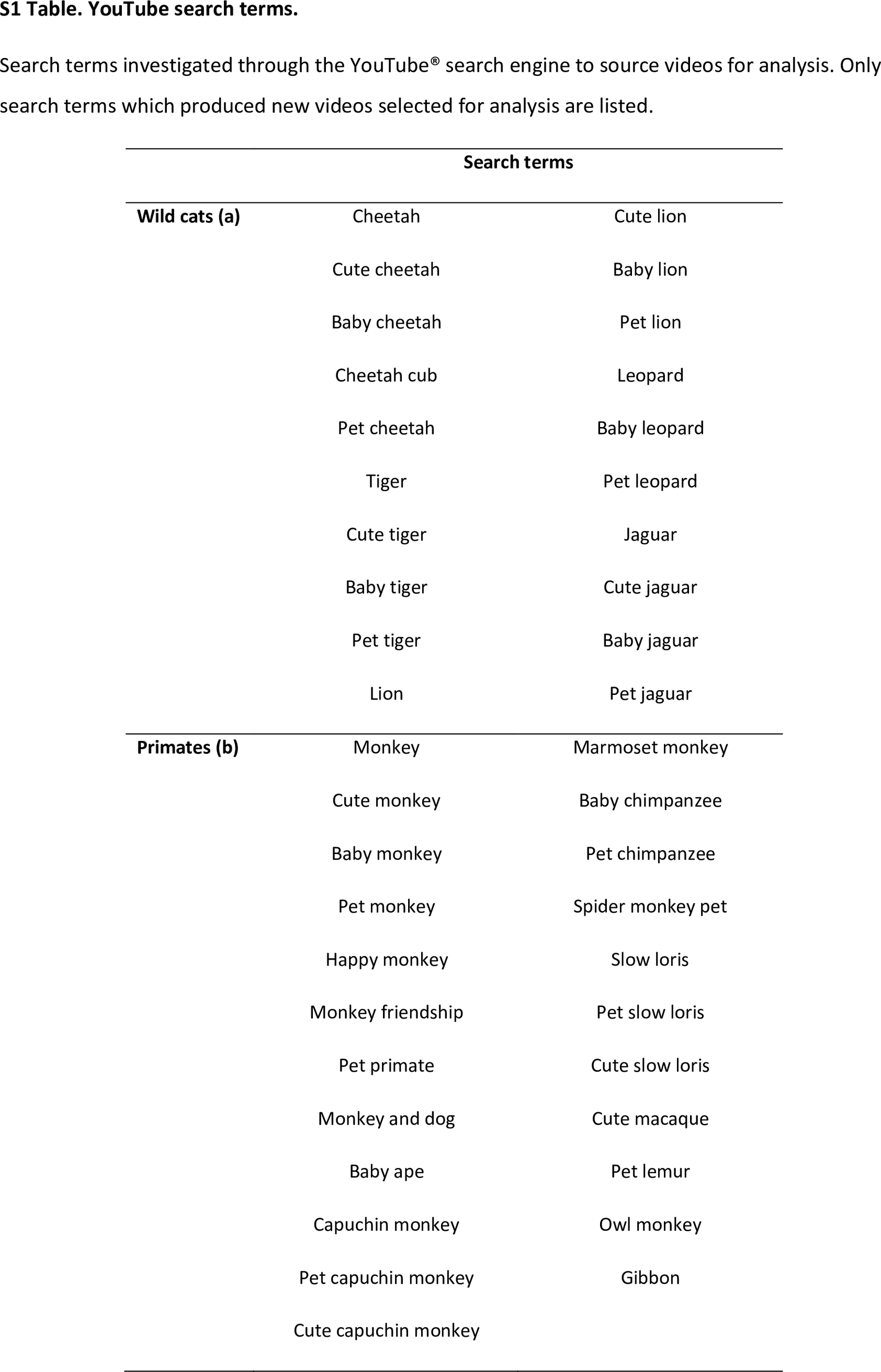

Supplement: S1 Table — Search terms investigated through the YouTube® search engine to source videos for analysis. Only search terms which produced new videos selected for analysis are listed. (TIF) [file pone.0235451.s001.tif]

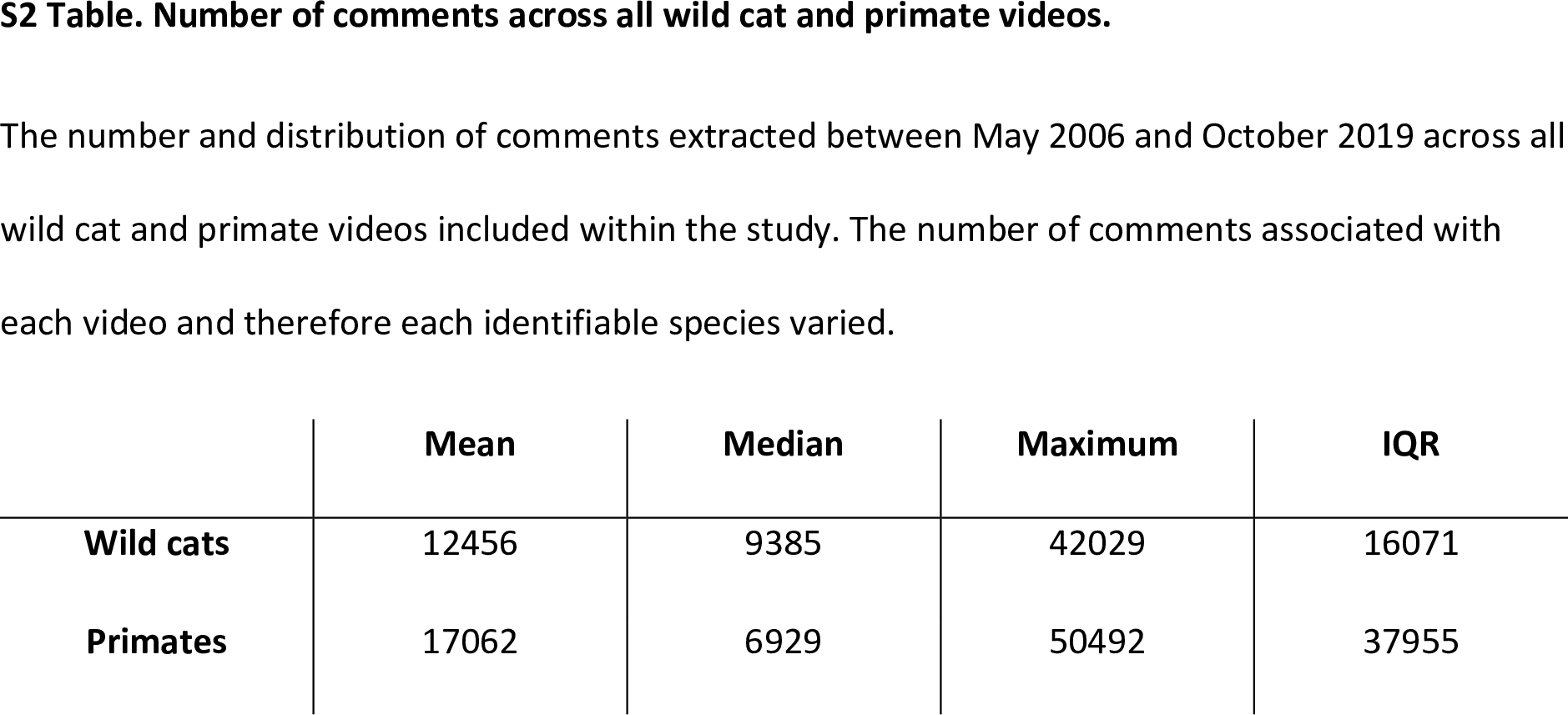

Supplement: S2 Table — The number and distribution of comments extracted between May 2006 and October 2019 across all wild cat and primate videos included within the study. The number of comments associated with each video and each identifiable species varied. (TIF) [file pone.0235451.s002.tif]

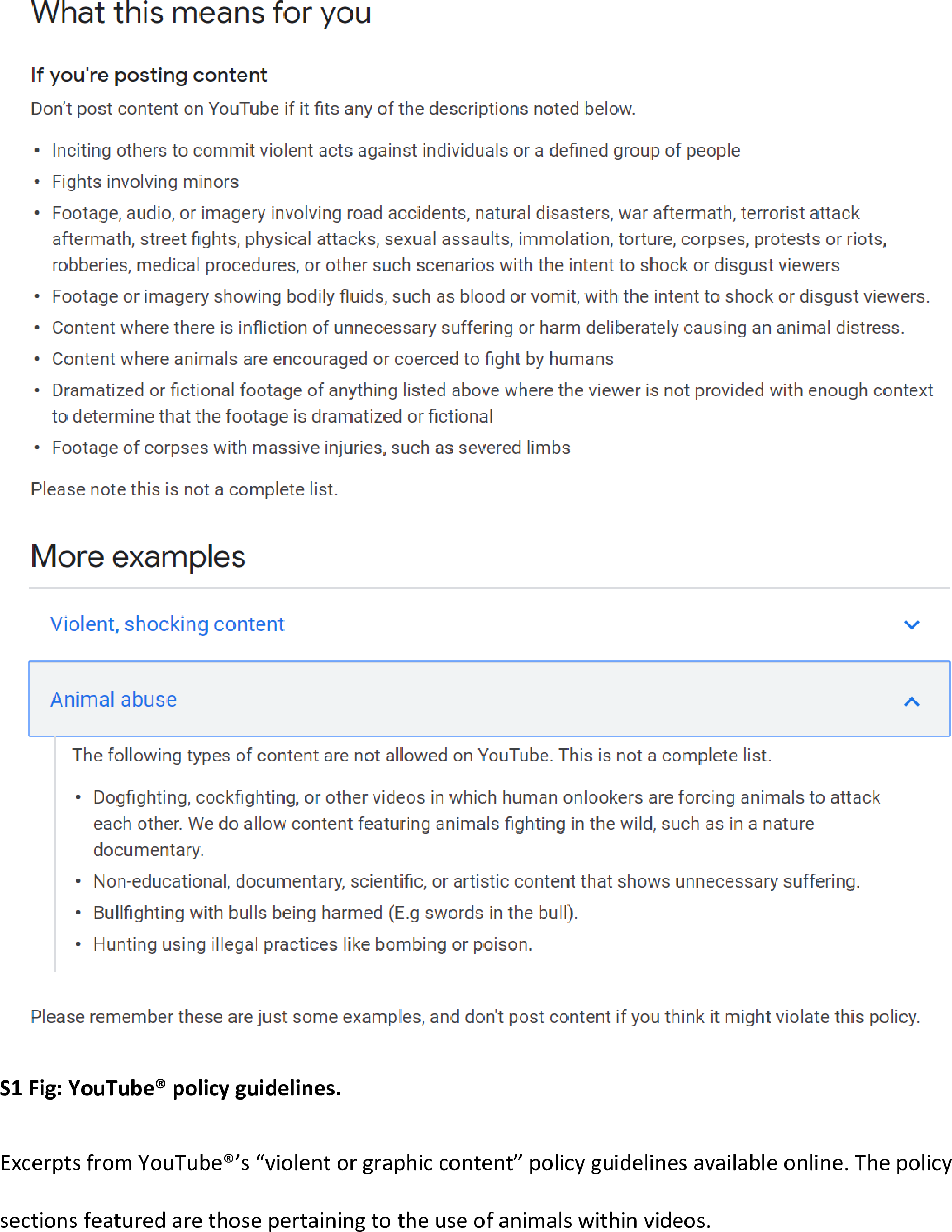

Supplement: S1 Fig — Excerpts from YouTube®’s “violent or graphic content” policy guidelines available online. The policy sections featured are those pertaining to the use of animals within videos. (TIF) [file pone.0235451.s004.tif]
