# Supplementary material for: Is YouTube promoting the exotic pet trade? Analysis of the global public perception of popular YouTube videos featuring threatened exotic animals
Source: PLoS One. 2021 Apr 13;16(4):e0235451. doi: 10.1371/journal.pone.0235451 (PMC8043400; doi:10.1371/journal.pone.0235451)
Supplement: S3 Table — The top ten most popular videos included within the study, according to view count at the time of data extraction, listed with primary species featured and additional video specifications. (PDF) [file pone.0235451.s003.pdf]

S3 Table. Popular videos within the study.

The top ten most popular videos included within the study, according to view count at the time of data extraction, listed with primary species featured and additional video specifications.

Rank	View count[1]	Country of origin	Species	Setting	Year of publication	IUCN[2]	Like:Dislike[3]
1	459940431	India	Macaque	Pet	2017	Endangered	1.8
2	253970575	USA	Capuchin	Pet	2017	Least Concern	2.5
3	64475478	Brazil	Tiger	Pet	2013	Endangered	7.1
4	55579835	South Africa	Lion	Captive	2015	Vulnerable	25.2
5	53046322	USA	Capuchin	Pet	2016	Least Concern	3.6
6	52528650	Australia	Tiger	Captive	2015	Endangered	19.3
7	44041015	Czech Republic	Lion	Pet	2014	Vulnerable	3.0
8	43843630	South Africa	Tiger	Pet	2016	Endangered	4.8
9	43595357	Vietnam	Macaque	Pet	2019	Endangered	3.1
10	40302702	USA	Tiger	Pet	2014	Endangered	20.8

[1]Number of views obtained at time of data extraction

[2]International Union for Conservation of Nature (IUCN, 2019)

[3]Likes and dislikes are defined by the rating system associated with videos on YouTube®, wherein viewers are able to either press the 'thumbs up' button to leave a like, or 'thumbs down' button to express dislike. The like:dislike ratio refers to the number of likes compared with dislikes added to videos by viewers up to the point of data extraction. A ratio of one would indicate equal likes and dislikes. The higher the ratio is above one, the more positively the video was received.
