# Supplementary material for: Is YouTube promoting the exotic pet trade? Analysis of the global public perception of popular YouTube videos featuring threatened exotic animals
Source: PLoS One. 2021 Apr 13;16(4):e0235451. doi: 10.1371/journal.pone.0235451 (PMC8043400; doi:10.1371/journal.pone.0235451)
Supplement: S1 Appendix — (PDF) [file pone.0235451.s005.pdf]

S1 Appendix

Exotic wild cat video URLs (n = 203)

1. https://www.youtube.com/watch?v=caX24UC2lsY
2. https://www.youtube.com/watch?v=er8-QSqRO7U
3. https://www.youtube.com/watch?v=DXTxOXNL2-8
4. https://www.youtube.com/watch?v=Ndlf5_L5gsE
5. https://www.youtube.com/watch?v=WTHDxJ_JWzY
6. https://www.youtube.com/watch?v=JlGfi2ofzFc
7. https://www.youtube.com/watch?v=cBz-1lEVS9M
8. https://www.youtube.com/watch?v=2btR9rAcdzo
9. https://www.youtube.com/watch?v=5XNdtZTyX2o
10. https://www.youtube.com/watch?v=EhrcbB-v-sU
11. https://www.youtube.com/watch?v=NTb2Cpd6ktE
12. https://www.youtube.com/watch?v=i3waAOcJkps
13. https://www.youtube.com/watch?v=ZJxXjyeUwZY
14. https://www.youtube.com/watch?v=EOY1LVjohrw
15. https://www.youtube.com/watch?v=PfwBOCxEst8
16. https://www.youtube.com/watch?v=pcOwnqBl2a0
17. https://www.youtube.com/watch?v=f7HcbjI52L0
18. https://www.youtube.com/watch?v=vmZzFwu5dLY
19. https://www.youtube.com/watch?v=uuaQ3tmIW0M
20. https://www.youtube.com/watch?v=g1uxy-ZcJBM&t=6s
21. https://www.youtube.com/watch?v=axcPoS2sF0E
22. https://www.youtube.com/watch?v=JmHGPC3lTUw
23. https://www.youtube.com/watch?v=JlGfi2ofzFc&t=4s
24. https://www.youtube.com/watch?v=2btR9rAcdzo
25. https://www.youtube.com/watch?v=caX24UC2lsY&t=149s
26. https://www.youtube.com/watch?v=BhbdWZXd2U0
27. https://www.youtube.com/watch?v=0tmCIsSpvC8
28. https://www.youtube.com/watch?v=er8-QSqRO7U&t=163s
29. https://www.youtube.com/watch?v=vmZzFwu5dLY
30. https://www.youtube.com/watch?v=uuaQ3tmIW0M&t=42s
31. https://www.youtube.com/watch?v=g04ipP_RqBE
32. https://www.youtube.com/watch?v=DXTxOXNL2-8&t=27s
33. https://www.youtube.com/watch?v=5XNdtZTyX2o&t=91s
34. https://www.youtube.com/watch?v=NTb2Cpd6ktE&t=263s
35. https://www.youtube.com/watch?v=ZJxXjyeUwZY&t=24s
36. https://www.youtube.com/watch?v=7jYPfOL6AuI
37. https://www.youtube.com/watch?v=pcOwnqBl2a0&t=27s
38. https://www.youtube.com/watch?v=caX24UC2lsY&t=123s
39. https://www.youtube.com/watch?v=_pl3Kk-K-eU
40. https://www.youtube.com/watch?v=Ht-tTZm0BzI
41. https://www.youtube.com/watch?v=er8-QSqRO7U&t=212s
42. https://www.youtube.com/watch?v=vmZzFwu5dLY
43. https://www.youtube.com/watch?v=mvsj3eJpjQk
44. https://www.youtube.com/watch?v=cUwzxVu6n08
45. https://www.youtube.com/watch?v=vlE8jED-x3k
46. https://www.youtube.com/watch?v=WTHDxJ_JWzY&t=262s

47. https://www.youtube.com/watch?v=cBz-1lEVS9M&t=8s
48. https://www.youtube.com/watch?v=JmHGPC3lTUw&t=27s
49. https://www.youtube.com/watch?v=HLCWwvd45sU
50. https://www.youtube.com/watch?v=xOZUbXKQITY
51. https://www.youtube.com/watch?v=_UbDeqPdUek
52. https://www.youtube.com/watch?v=21ZEIbjY9VI
53. https://www.youtube.com/watch?v=is0Z5cryqcM
54. https://www.youtube.com/watch?v=xmZw6ejvabU
55. https://www.youtube.com/watch?v=8pGTgkcP-RU
56. https://www.youtube.com/watch?v=1J0C2HlC38c
57. https://www.youtube.com/watch?v=yndqQARnsTU
58. https://www.youtube.com/watch?v=HulXj2UHm3M
59. https://www.youtube.com/watch?v=swtPVeyDJIl
60. https://www.youtube.com/watch?v=EdCVijVT7Wk
61. https://www.youtube.com/watch?v=igQRWZJklIo
62. https://www.youtube.com/watch?v=RPekEt1Kjg8
63. https://www.youtube.com/watch?v=tvID7SqH4wA
64. https://www.youtube.com/watch?v=fcRRJHVtGPw
65. https://www.youtube.com/watch?v=nUCiSHwgHj8
66. https://www.youtube.com/watch?v=3e5u9gX1fWM
67. https://www.youtube.com/watch?v=tklx3j7kgJY
68. https://www.youtube.com/watch?v=4hvpUsYG3PI
69. https://www.youtube.com/watch?v=ZEbJV61L2r0
70. https://www.youtube.com/watch?v=3IUeFj7Iyw4
71. https://www.youtube.com/watch?v=1vUuHqqTQTw
72. https://www.youtube.com/watch?v=nJQwfOFMGSI
73. https://www.youtube.com/watch?v=57fSN4Ee0ho
74. https://www.youtube.com/watch?v=EQ_4dicv7aQ
75. https://www.youtube.com/watch?v=DfHARgviUzQ
76. https://www.youtube.com/watch?v=g8scRVtG1ag
77. https://www.youtube.com/watch?v=x7Bo0wleT7o
78. https://www.youtube.com/watch?v=K4bESmTQe5s
79. https://www.youtube.com/watch?v=kt7u99p68Aw
80. https://www.youtube.com/watch?v=D43u9kMl2qM
81. https://www.youtube.com/watch?v=BWxNXC8Tvo8
82. https://www.youtube.com/watch?v=B5We-5c9OX0
83. https://www.youtube.com/watch?v=gekQ2nfzZfQ
84. https://www.youtube.com/watch?v=sMnK63ppBXU
85. https://www.youtube.com/watch?v=BVfJufGs54U
86. https://www.youtube.com/watch?v=gRI3ijc8xvk
87. https://www.youtube.com/watch?v=xwidefc2wpc
88. https://www.youtube.com/watch?v=DrRqkW2jkF0
89. https://www.youtube.com/watch?v=idRc_KkInds
90. https://www.youtube.com/watch?v=dcs-H5p-Myw
91. https://www.youtube.com/watch?v=EEtVsx5xVos
92. https://www.youtube.com/watch?v=Tdjk9dhT_AU
93. https://www.youtube.com/watch?v=jVOTo18R8_A
94. https://www.youtube.com/watch?v=zLq119PZV9k

95. https://www.youtube.com/watch?v=VjHSfnDCxvM
96. https://www.youtube.com/watch?v=CxVP2ySWb-o
97. https://www.youtube.com/watch?v=LBBuVxXotGg
98. https://www.youtube.com/watch?v=3aBJ-CJPnRQ
99. https://www.youtube.com/watch?v=fJnorMM97AI
100. https://www.youtube.com/watch?v=rmpwJdzIEgI
101. https://www.youtube.com/watch?v=K9ayDGf8jVk
102. https://www.youtube.com/watch?v=aHz6XOA08xQ
103. https://www.youtube.com/watch?v=4iPq5CbGW6c
104. https://www.youtube.com/watch?v=7MwQYcY479o
105. https://www.youtube.com/watch?v=HvVXyOj9wl8
106. https://www.youtube.com/watch?v=78i8ecrVnqk
107. https://www.youtube.com/watch?v=K9Pd46v1lno
108. https://www.youtube.com/watch?v=NN3vFfyH4-E
109. https://www.youtube.com/watch?v=t9je3P-_NmU
110. https://www.youtube.com/watch?v=qd4i3J5GbHI
111. https://www.youtube.com/watch?v=YtaLLkHwMi4
112. https://www.youtube.com/watch?v=8SDdSsAm4K4
113. https://www.youtube.com/watch?v=ljWlOCWiNIA
114. https://www.youtube.com/watch?v=smYZ7nJp5wg
115. https://www.youtube.com/watch?v=muzX_0SBFio
116. https://www.youtube.com/watch?v=wLEMvkrIdnA
117. https://www.youtube.com/watch?v=11cClvR1e3U
118. https://www.youtube.com/watch?v=eUg9E41ImzY
119. https://www.youtube.com/watch?v=Rwv8mCw8JJs
120. https://www.youtube.com/watch?v=VBJvkSvahpw
121. https://www.youtube.com/watch?v=3TmSwF0mejg
122. https://www.youtube.com/watch?v=DRQ2Ie7cIes
123. https://www.youtube.com/watch?v=KLdHheUhaeo
124. https://www.youtube.com/watch?v=lwMybWFtQh8
125. https://www.youtube.com/watch?v=7HtpR439m-w
126. https://www.youtube.com/watch?v=Znpnf9D2VIk
127. https://www.youtube.com/watch?v=BZT1dIqXHLU
128. https://www.youtube.com/watch?v=Wn_bMZQ60cY
129. https://www.youtube.com/watch?v=-H5PhdpOKhY
130. https://www.youtube.com/watch?v=JWphg9uluFM
131. https://www.youtube.com/watch?v=7z8KLlCfzwc
132. https://www.youtube.com/watch?v=_e0LQslOiYk
133. https://www.youtube.com/watch?v=J8Ww9sdgOjk
134. https://www.youtube.com/watch?v=UAPf_hRoRtM
135. https://www.youtube.com/watch?v=dfsgvjsrptI
136. https://www.youtube.com/watch?v=kP53H4Gi6W8
137. https://www.youtube.com/watch?v=RB5_HZWf49E
138. https://www.youtube.com/watch?v=2MUAOStqrCc
139. https://www.youtube.com/watch?v=nv2O0GahNtM
140. https://www.youtube.com/watch?v=9Ciyd4AggzI
141. https://www.youtube.com/watch?v=2f8Hpid8YWA
142. https://www.youtube.com/watch?v=IlXjoPtTGT8

143. https://www.youtube.com/watch?v=zVNTdWbVBgc
144. https://www.youtube.com/watch?v=NT1HGnotQWc
145. https://www.youtube.com/watch?v=btuxO-C2IzE
146. https://www.youtube.com/watch?v=QBjKntYuUIk
147. https://www.youtube.com/watch?v=ghvCGSQ7mFw
148. https://www.youtube.com/watch?v=JPooopPSlD8
149. https://www.youtube.com/watch?v=6fbahS7VSFs
150. https://www.youtube.com/watch?v=zUbZk6Gf-JA&t=601s
151. https://www.youtube.com/watch?v=FCFUC6KqQJo
152. https://www.youtube.com/watch?v=VJgsmr92udc
153. https://www.youtube.com/watch?v=GrsT-hDQVsg
154. https://www.youtube.com/watch?v=mhe9YYI1HsE
155. https://www.youtube.com/watch?v=IChRNbuHHWE
156. https://www.youtube.com/watch?v=aukIXtPvVXE
157. https://www.youtube.com/watch?v=GzG7TEvUGn8
158. https://www.youtube.com/watch?v=-b-VQI5smMI
159. https://www.youtube.com/watch?v=fykC8TRtknQ
160. https://www.youtube.com/watch?v=vZp2RyiHIuY
161. https://www.youtube.com/watch?v=XPinz3wwMN8
162. https://www.youtube.com/watch?v=8J8-M09R7Lw
163. https://www.youtube.com/watch?v=UFoEdgsAr7Y
164. https://www.youtube.com/watch?v=cv901tRE0rQ
165. https://www.youtube.com/watch?v=7cM4jj5UiuQ
166. https://www.youtube.com/watch?v=qWATnuZWQ58
167. https://www.youtube.com/watch?v=6SKh9iPWu1Y
168. https://www.youtube.com/watch?v=tU9pkFsdYpg
169. https://www.youtube.com/watch?v=ZR6zPp96xNI
170. https://www.youtube.com/watch?v=y-zGH-7R2G8
171. https://www.youtube.com/watch?v=9NztY2bo8H0
172. https://www.youtube.com/watch?v=SnjizeGokRg
173. https://www.youtube.com/watch?v=ViJV_EwmJU8
174. https://www.youtube.com/watch?v=RPEPGF6oCMc
175. https://www.youtube.com/watch?v=x8co8ARw2BU
176. https://www.youtube.com/watch?v=z4FD4ho_XiE
177. https://www.youtube.com/watch?v=N_uVsmSbgCE
178. https://www.youtube.com/watch?v=QR44f2SRWGE
179. https://www.youtube.com/watch?v=iox86q2JuHA
180. https://www.youtube.com/watch?v=OHRQ9XwIsnE
181. https://www.youtube.com/watch?v=Co3N4-6B2pM
182. https://www.youtube.com/watch?v=d-0DozSG5oo
183. https://www.youtube.com/watch?v=xYLjl9qGBhA
184. https://www.youtube.com/watch?v=axcPoS2sF0E&t=40s
185. https://www.youtube.com/watch?v=E4MUXs4IHtY
186. https://www.youtube.com/watch?v=NWuFoPguAlE
187. https://www.youtube.com/watch?v=wYr_5gRMFRU
188. https://www.youtube.com/watch?v=_88PFzWFDxo
189. https://www.youtube.com/watch?v=wZjZL3S3mTg
190. https://www.youtube.com/watch?v=X4N936oDeWI

191. https://www.youtube.com/watch?v=tITXiO_g4Bw
192. https://www.youtube.com/watch?v=XOfuJY3uvug
193. https://www.youtube.com/watch?v=Q2arqnpQphk
194. https://www.youtube.com/watch?v=UdwRo9wfKmc
195. https://www.youtube.com/watch?v=UWkIMUfamxI
196. https://www.youtube.com/watch?v=BodIRbaY93Y
197. https://www.youtube.com/watch?v=Vcxvzvokae0
198. https://www.youtube.com/watch?v=DrjmHYiWUc0
199. https://www.youtube.com/watch?v=jXsdKpOWL1U
200. https://www.youtube.com/watch?v=nPMi136tURg
201. https://www.youtube.com/watch?v=yXjwNF-tkEM
202. https://www.youtube.com/watch?v=3C-Q8n_j8ZA
203. https://www.youtube.com/watch?v=XqQwQzA9XU4

Primate video URLs (n = 143)

1. https://www.youtube.com/watch?v=xjodBEJpA-Q
2. https://www.youtube.com/watch?v=giV3NOIXZQo
3. https://www.youtube.com/watch?v=lPmOcJ9YdYw
4. https://www.youtube.com/watch?v=spMkaJp975s
5. https://www.youtube.com/watch?v=vf9osFdrdVo
6. https://www.youtube.com/watch?v=eH_NEGnHqzo&t=77s
7. https://www.youtube.com/watch?v=nmiCF9aTMKY&t=437s
8. https://www.youtube.com/watch?v=pvt1nrfI0YM
9. https://www.youtube.com/watch?v=LCNugP6TkaQ
10. https://www.youtube.com/watch?v=1GL35T21k54
11. https://www.youtube.com/watch?v=e1THKmao7gk
12. https://www.youtube.com/watch?v=0_9Gj1kIhHY
13. https://www.youtube.com/watch?v=KQFK4crc9Fg
14. https://www.youtube.com/watch?v=AbK9q9XO26Q
15. https://www.youtube.com/watch?v=DLB13vQNpvg
16. https://www.youtube.com/watch?v=oMsxViJ3-oQ
17. https://www.youtube.com/watch?v=grkLBFR1cOw
18. https://www.youtube.com/watch?v=_Nvox6ke7ZQ
19. https://www.youtube.com/watch?v=OLFCxeubBHY
20. https://www.youtube.com/watch?v=hOK3LIiC2Bs
21. https://www.youtube.com/watch?v=3LtBpC5jpSs
22. https://www.youtube.com/watch?v=FWwBQk_jhMY
23. https://www.youtube.com/watch?v=bAgaKNl3wPY
24. https://www.youtube.com/watch?v=T9897fMrOS0
25. https://www.youtube.com/watch?v=WWjA2aSnLnU
26. https://www.youtube.com/watch?v=OGdU1Kh4Rj8
27. https://www.youtube.com/watch?v=kGhREWgofMI
28. https://www.youtube.com/watch?v=778xtRCYwoE
29. https://www.youtube.com/watch?v=g6ZM2Q-D0b8
30. https://www.youtube.com/watch?v=a18ac1dwt8s
31. https://www.youtube.com/watch?v=V0Gzi-SIj6Y
32. https://www.youtube.com/watch?v=ZaUtSe9wwPc

33. https://www.youtube.com/watch?v=ZyCjHGVBIwg
34. https://www.youtube.com/watch?v=b6m-XlOxjbk
35. https://www.youtube.com/watch?v=ToaSaVLWnds
36. https://www.youtube.com/watch?v=_6iN3YGHEEs
37. https://www.youtube.com/watch?v=FATKDfkfecI
38. https://www.youtube.com/watch?v=0_OoS9bzsgY
39. https://www.youtube.com/watch?v=L0eTgkdIc0c
40. https://www.youtube.com/watch?v=ToCWKsxPkZY
41. https://www.youtube.com/watch?v=FBDHGcofh6U
42. https://www.youtube.com/watch?v=WTY9pWETrhA&t=518s
43. https://www.youtube.com/watch?v=QuNfBba6IhE
44. https://www.youtube.com/watch?v=GGz-MM1gT3s
45. https://www.youtube.com/watch?v=jf9OFkVj0DE
46. https://www.youtube.com/watch?v=AnFUGyvclOw
47. https://www.youtube.com/watch?v=p-nN0UDY-xY
48. https://www.youtube.com/watch?v=NuYjJXMnYx8
49. https://www.youtube.com/watch?v=Q7g_ektnyyY
50. https://www.youtube.com/watch?v=rcLxqQmaeco
51. https://www.youtube.com/watch?v=9pKYIN0HbzI&t=112s
52. https://www.youtube.com/watch?v=Fa1-FzHzQFI
53. https://www.youtube.com/watch?v=eXdeOag5LYI
54. https://www.youtube.com/watch?v=4jF4309UgY4
55. https://www.youtube.com/watch?v=1wnEkpHD64U
56. https://www.youtube.com/watch?v=xaY9XOjZwHc
57. https://www.youtube.com/watch?v=FBCM8-24TyE
58. https://www.youtube.com/watch?v=14FAl-YiA1U
59. https://www.youtube.com/watch?v=ZYaxg5NZW_g
60. https://www.youtube.com/watch?v=J4kfC_jwZyc
61. https://www.youtube.com/watch?v=AFIup7uCKw4
62. https://www.youtube.com/watch?v=HXmOd0Ew0QI
63. https://www.youtube.com/watch?v=1K-eOz_-uww
64. https://www.youtube.com/watch?v=PZ5ACLVjYwM
65. https://www.youtube.com/watch?v=2Th199ySVdU
66. https://www.youtube.com/watch?v=2Syd_BUbl5A
67. https://www.youtube.com/watch?v=K9s5xYPsG8c
68. https://www.youtube.com/watch?v=zpV7L--cQ8s
69. https://www.youtube.com/watch?v=z4n60LOeY-U
70. https://www.youtube.com/watch?v=5_sfnQDr1-o
71. https://www.youtube.com/watch?v=lGNobvJwZbI
72. https://www.youtube.com/watch?v=ZhazCS0CTpQ&t=11s
73. https://www.youtube.com/watch?v=KYfmzbZKbKA
74. https://www.youtube.com/watch?v=pG9sNjdvj_g
75. https://www.youtube.com/watch?v=LoITllrQdrU
76. https://www.youtube.com/watch?v=XrI1-1fseRQ
77. https://www.youtube.com/watch?v=F1jMkXfjaL4
78. https://www.youtube.com/watch?v=pamvVgkN1Rk
79. https://www.youtube.com/watch?v=Q98oGy_FyO0
80. https://www.youtube.com/watch?v=FLVtv3nTeG0

81. https://www.youtube.com/watch?v=MDStH49W5Hk
82. https://www.youtube.com/watch?v=f0xuDPi-9oU
83. https://www.youtube.com/watch?v=buWEZ8Dpi_E
84. https://www.youtube.com/watch?v=dGkankqyCX8
85. https://www.youtube.com/watch?v=BYNoQZ5djUA
86. https://www.youtube.com/watch?v=nU6c3pcqrLE
87. https://www.youtube.com/watch?v=X90n1bT1a1E
88. https://www.youtube.com/watch?v=oppWVrPpkX8
89. https://www.youtube.com/watch?v=CxcDsa5GJQE
90. https://www.youtube.com/watch?v=uFDPLyAWpVg
91. https://www.youtube.com/watch?v=8PtPCCGDxxo
92. https://www.youtube.com/watch?v=vb6CLHbP7vY
93. https://www.youtube.com/watch?v=kCAiZmzdP-s
94. https://www.youtube.com/watch?v=6GsK4xF6hnk
95. https://www.youtube.com/watch?v=nzeRGGXcXcw
96. https://www.youtube.com/watch?v=Oq4CS-zomek
97. https://www.youtube.com/watch?v=S5EwG0iccJA
98. https://www.youtube.com/watch?v=2_AHpR6bsvs
99. https://www.youtube.com/watch?v=E8-qxBINl28
100. https://www.youtube.com/watch?v=ArSG0Wnj828
101. https://www.youtube.com/watch?v=xUMQbc26UHM
102. https://www.youtube.com/watch?v=Ws6Swr8R2Kg
103. https://www.youtube.com/watch?v=kX0kAtUrJFY
104. https://www.youtube.com/watch?v=OMl51TySbY0
105. https://www.youtube.com/watch?v=KHtocSC7hRU
106. https://www.youtube.com/watch?v=ANzH3EOd9yA
107. https://www.youtube.com/watch?v=ZwfrNqwVCPQ
108. https://www.youtube.com/watch?v=PFU30fJeIw8
109. https://www.youtube.com/watch?v=KdBKM6DrSHQ
110. https://www.youtube.com/watch?v=1_5C8u5QV5Q
111. https://www.youtube.com/watch?v=k-uglAo4IT8
112. https://www.youtube.com/watch?v=EjogSyxPL0o
113. https://www.youtube.com/watch?v=hW2M8vX94D8
114. https://www.youtube.com/watch?v=WBqtu1Qc5a8
115. https://www.youtube.com/watch?v=dTqGclS-VWA
116. https://www.youtube.com/watch?v=oWZKSiSDzv0
117. https://www.youtube.com/watch?v=nPmVSPQykoc
118. https://www.youtube.com/watch?v=XggFmDS5qIc
119. https://www.youtube.com/watch?v=6lA7u6fTXw8
120. https://www.youtube.com/watch?v=4GN5BbdotlI
121. https://www.youtube.com/watch?v=OgJbG-xUrrA
122. https://www.youtube.com/watch?v=IV-Y6tPZNdk
123. https://www.youtube.com/watch?v=unW-Pgb8Q8I
124. https://www.youtube.com/watch?v=HPCVL6redvU
125. https://www.youtube.com/watch?v=sPIhTQ8x1tw
126. https://www.youtube.com/watch?v=puEDDhrivzY
127. https://www.youtube.com/watch?v=oFBu0jmTQ6Y
128. https://www.youtube.com/watch?v=BW10g1ZS6wo

129. https://www.youtube.com/watch?v=wcEK-K7ysTc
130. https://www.youtube.com/watch?v=-twAZM4Sw84
131. https://www.youtube.com/watch?v=01ZZGdOzEv8
132. https://www.youtube.com/watch?v=Qc2hlHDvNjk
133. https://www.youtube.com/watch?v=EM_EsFzolAU
134. https://www.youtube.com/watch?v=YXi2j6WfTlI
135. https://www.youtube.com/watch?v=RCJaFlyz_gU
136. https://www.youtube.com/watch?v=xlJqzhmD02k
137. https://www.youtube.com/watch?v=Qc2hlHDvNjk
138. https://www.youtube.com/watch?v=glX01OtG2vo
139. https://www.youtube.com/watch?v=CdGV6JxXy9s
140. https://www.youtube.com/watch?v=_SNE2MYAotU
141. https://www.youtube.com/watch?v=PnC5TTqPrxM
142. https://www.youtube.com/watch?v=9-kZY_BeNIo
143. https://www.youtube.com/watch?v=jcFbWPRXn1w

Selected videos (published between May 2006 and October 2019) were available for public viewing online and the use of this dataset was in compliance with the YouTube® Terms of Service. Please note that some video statistics and comment availability have changed since the point of data collection for this study (17 June 2019 – 4 October 2019).
