# Supplementary material for: Is YouTube promoting the exotic pet trade? Analysis of the global public perception of popular YouTube videos featuring threatened exotic animals
Source: PLoS One. 2021 Apr 13;16(4):e0235451. doi: 10.1371/journal.pone.0235451 (PMC8043400; doi:10.1371/journal.pone.0235451)
Supplement: Attachment — Submitted filename: D-20-17651_review.pdf [file pone.0235451.s006.pdf]

Review for *Is YouTube promoting the exotic pet trade? Analysis of the global public perception of popular YouTube videos featuring threatened exotic animals.*

In this paper the authors collected comments from YouTube videos to assess sentiment towards threatened exotic animals. The number of comments and videos the authors compiled is impressive. I also like the large range of species the authors collected data from. I do, however, have some concerns with this research. These concerns are related to how the authors are interpreting their analysis and how they analyzed their data. More specifically:

1. I think the authors could be a little more careful about what the data coming out of an analysis of YouTube comments provides. Perceptions, beliefs, and attitudes all mean something very specific in the social science literature, and analyzing single comments from YouTube users is not likely a way to understand a users perception towards or attitudes about a given topic, which are certainly more complex than what can be assessed from a singular comment. Discussing these comments as "opinions" may be a more careful interpretation of the data that is available.

2. While the application of sentiment analysis to YouTube comments is promising, the authors treatment of these data is lacking. From my reading of the methods and the results is appears that the authors only descriptively compared means of the values derived from the sentiment analysis. A more thorough statistical treatment of these data is absolutely necessary. Some potential issues that I see:

    1. The content of a video likely has an influence on the comments made, and the number of comments likely varies among videos. Accounting for unequal sample-sizes among videos and quantifying the variability among videos for each species is important. Depending on the response variable, a generalized linear mixed model or linear mixed model could be used to account for this.

    2. I bring this up a bit in the results, but the authors stated that they collected videos of these species in captivity as well as videos of them as exotic pets. I did not see any comparison of this, and I would imagine that this would be absolutely necessary. Likewise, zoos can vary substantially (accredited institutions to road-side attractions and therefore the general 'zoo' category may not be that applicable).

3. I'm a little uncertain about the Emoji analysis. First, there should be some description about the extra information gained from incorporating emojis. Second, the results shared about this is about 2 sentences long (line 186 - 189). I imagine that there is more details the authors could potentially share here if the emoji details provide us with some additional nuanced understanding of this topic, especially at they collected a substantial amount of data across species.

Below I've provided substantial comments on each section of the manuscript. They are organized into top-level thoughts for each section and then line by line comments. I hope that the authors find them useful.

Introduction

Top-level thoughts

1. I think one of the key points that the authors need to make within the first couple paragraphs of the introduction is about the link between social media and the exotic pet trade. Right now, the current link is implicitly made but I think it would be much stronger to be more explicit with this relationship. By the time that the authors arrive at YouTube, it would make sense to cement the idea that a) the exotic pet trade threatens biodiversity b) that public perception towards exotic pets plays a big part in this and c) social media can not only influence public perception, but it's also a major platform for people to 'interact' with exotic pets. However, since content is often not regulated this could make things worse.

A lot of this information exists in the first couple paragraphs, but with a little bit of rewriting the authors could really set the foundation for why we need to assess comments on YouTube.

2. For the introduction to make sense a reader needs to know that there are videos of exotic pets (or animals in general) up on YouTube. I'd try making that more clear in the third paragraph.

3. The authors need to spend a little more time developing how sentiment analysis can help them achieve their research objectives. Part of this is explaining to a reader what sentiment analysis is, but also to link back to how it can be a useful tool in this specific instance.

Line by line comments

Line 32: ..."important challenge to conserve species" instead of "...important challenge to maintaining species conservation."

Line 32 - 34: This is just a personal preference, but I think the opening could be a little more direct and have more impact by removing some of the passive voice. Is it important to say that unsustainable trade has been recognized as an important challenge or that is an important challenge? Likewise, is it important to say that exotic pets have been defined as... or to just say that Exotic pets are...?

Line 32 - 39: This opening paragraph lacks a resolution. I think it could help to add one extra sentence at the end that distills down some of this information into a key takeaway point.

Line 40 - 41: What is the trade? I'm assuming the authors mean exotic pet trade. Additionally, as the last paragraph was about the exotic pet trade it might make more sense to start this sentence about the exotic pet trade instead of social media (i.e., the flow between these paragraphs could be improved a little).

Line 42: What do the authors mean by "such platforms"? Maybe it would be more clear to simply say "Social media platforms..."?

Line 40 - 47: This paragraph is also missing a closing point. Why does it matter that social media content is unregulated and how does it relate back to the exotic pet trade? As it stands, the link between the exotic pet trade and social media could be made much stronger.

Line 51: What do the authors mean by "insufficiently regulated"?

Line 48 - 60: The topic sentence of this paragraph is about YouTube but most of the content of the paragraph is about the exotic pet trade and how it may be possible to investigate public perception towards the exotic pet trade. A new topic sentence that briefly introduces the overall subject of this paragraph would greatly improve clarity.

Line 61 - 64: This paragraph follows a common mistake I see in science papers. Instead of saying "we did X to learn Y" (i.e., we used sentiment analysis to learn Y) it makes a lot more sense to flip this around (to learn X we did Y). Present the question and then lay out the approach to answering it.

Line 64 - 66: This comes out of the blue. I'd probably put some of this info a little bit earlier in the intro and then remind the reader that this is why you focused on them.

Methods

Top-level comments

1. Some of the description in the methods needs a little more information, this is especially true for the lexicons used in the sentiment analysis. Why are the specific lexicons used to understand opinions towards these videos?

2. How were differences among categories assessed? The authors have explained how they generated the descriptive statistics from comments (i.e., how they did their sentiment analysis) but have yet to describe how they analyzed the resulting data.

Line by line comments

Line 90: R studio is just a graphical user interface, not a programming language. I'd rephrase to "Data management and analyses were conducted in R v. 3.6.1 (citation)"

Line 91: Can all of the comments be extracted via `tuber`? Looking at the package it seems like `tuber::get_comments()` can only pull 100 comments max. A little more info here would be helpful.

Line 109-111: What does -1, 0, and 1 mean for this sentiment analysis? Likewise, why were the afinn and bing lexicons used?

Line 118: R not R studio (see above).

Line 120 - 121: Why was this emoji sentiment lexicon used and how was it based on Novak et al (i.e., if there were some changes made please describe them).

Results

Top-level comments

1. The authors stated that they collected data on whether the videos were of animals in care of zoos or if they were exotic pets. However, this information is not presented. I would think that this could have a very substantial influence on how people perceive the subjects of a video. For each species, the proportion of videos of that species in captivity should be provided (e.g., in Table 1).

2. How many comments per video? If a user made multiple comments were they all incorporated? What about comments from the person who shared the video?

Line by line comments

Table 1: What do the column headers mean, frequency of what? Percentage of what, etc. The Table legend should provide this information.

Line 155 - 160: A lot of this is methodological information and should be moved up a section.

Line 192 - 194: How does this imply that there is a positive public perception? The mean and the variance of this estimate are important. For example, Normal(mu = 10, sd = 1) is very different from Normal(mu = 10, sd = 1000). I encourage the authors to apply some sort of statistical test to these data to provide some more evidence of these claims.

Line 186: How were the 10,000 comments selected across videos? Likewise, the methods did not describe any sub-sampling.

Line 186 - 189: This section is lacking.

Line 192 - 193: Comments made likely do not represent public perception.

Discussion

Top-level thoughts

1. Personally, I don't know what the additional headers in the discussion adds to it's flow, and from my reading I think it actually decreases the general flow of the discussion. Part of this issue could be from some of the general layout of the manuscript. The goals of the paper itself are quite vague. With some tweaking to the introduction and a clear description of what the authors are trying to learn then the flow of the discussion should improve. Why, for example, do we need to analyze comments over time, what insight does that gain us for the topic at hand?

2. A lot of the discussion serves as a review of other work but does not especially bring up the authors results in relation to this other research. As a result, the work the authors put together for their analysis is getting hidden behind all of the other information that is being presented within the discussion.

Line by line comments

Line 225: How are comments made on YouTube representative of public perception?

Line 225 - 230: This lead in to the discussion is not likely necessary. With some rewriting the authors could simply move straight into the discussion.

Line 233 - 234: This was not shared in the results. If understanding the frequency of specific words is important than the authors should move some of these quantitative bits to the results and leave the interpretation of these frequencies to the results.

Line 233 - 241: Explaining some of the limitations of sentiment analysis is absolutely necessary, but I think the authors need to more critically consider how this may be influencing their results. Likewise, how does the addition of using emojis help correct for sarcasm? Would it be possible to read a subset of comments for sarcasm to get an idea for how prevalent this in the authors data?

Line 304 - 331: Much of the changes the authors are suggesting here are top-down, in that they are encouraging YouTube to change their policies. Are there bottom up approaches that could work as well? I'm thinking about projects such as Wildbook, which uses machine learning to identify unique individuals of a species (they have a bot that will comment on a video if and ask for additional information if the video is of interest). Could a similar approach be useful here? What if there was an automated way to find videos of species of concern (especially with people in the videos) and provide additional information to users in the comments?

Figures

Figure 2

Increase font so that it is more readable on the axes. I'd probably order the IUCN categories in a way that goes from least concern to endangered as well (instead of alphabetical).

Figure 3

Same comments as Figure 2

Figure 4

Increase point size of dots on plot, why were emojis only available after 2014? Increase font so that it is more readable on the axes.

Figure 5

Same comments as Figure 4.
