# Supplementary material for: Is YouTube promoting the exotic pet trade? Analysis of the global public perception of popular YouTube videos featuring threatened exotic animals
Source: PLoS One. 2021 Apr 13;16(4):e0235451. doi: 10.1371/journal.pone.0235451 (PMC8043400; doi:10.1371/journal.pone.0235451)
Supplement: Attachment — Submitted filename: Response to Reviewers.pdf [file pone.0235451.s007.pdf]

Response to the Reviewers

Reviewer 1

Thank you for your comments and editing suggestions.

This research project was designed as a descriptive observational study and as such no hypothesis or research questions were developed. We wanted to explore whether there was a relationship between the variables, however as there is limited published research surrounding how the public respond to captive wildlife featured in online videos it seemed unreasonable to formulate a hypothesis as we did not know what to expect. Due to the gap in the literature, the paper was predominately a descriptive analysis. Further studies have been planned where data will be collected based on hypotheses.

1. In accordance with research conducted by Nekaris et al. add others, the 'cute factor' is commonly used as a marketing tool for pets, both domestic and exotic, as it increases the likelihood that a consumer will acquire a pet. The link between the 'cute' factor and the desire for an animal has been well established, therefore we evaluated the use of 'cute' and additional positive sentiment terms to suggest a higher likelihood that these people wanted the animals shown as a pet, not necessarily to say that they would be able to attain one. These positive terms were also associated with the keywords used to select videos. The exotic pet trade is fuelled by such desire and therefore the authors considered it appropriate to suggest a link between the depiction of 'cute' animals on YouTube® with increased demand for exotic pets. We have included a more thorough description of this point in line 279-281. Although it is a valid point, full analysis of the content itself, as suggested, was not part of the study and could not be accurately assessed based on our data.

2. The assessment of animal welfare was initially considered in the early stages of the research. However, upon analysing videos for the study, it was not possible to accurately assess the welfare state consistently across all videos as they were generally too short and the content disallowed the conditions to be assessed properly as certain aspects were not shown on screen. Therefore, it was difficult to accurately comment scientifically on welfare status, as mentioned in line 327-330.

3. Thank you for your comment. A statement has been added to the paper to enhance clarity regarding emoji availability. In regards to the macaque conservation status, not all macaque species were readily identifiable due to limitations imposed by video quality and duration. The identifiable macaques were classified as either Vulnerable or Endangered on the IUCN Red List, hence macaques in the study were generally represented as being 'Endangered' to reflect the most severe classification, as explained within the methods (line 101-104).

4. In regards to the seemingly random results regarding human interaction, the authors have edited the discussion to clarify why this was important. Human interaction, either directly seen within the video or implied as the animals were captive, was an important component of the study as we aimed to analyse videos showing animals involved in 'free handling' situations (see the discussion for further information on this, line 311-315) and to stress the importance of zoonoses (where the discussion was edited to highlight this, as suggested). Thank you for your suggestion to clarify this point and to link the discussion back to the zoonosis risks, this has been considered and hopefully explained more clearly in the revised discussion.

In regards to the grammar check, we thank you for bringing this to our attention. The paper was edited carefully to try to overcome this.

Reviewer 2
Thank you for your time and comments.

In terms of how we tokenised the text, we decided to use 1-grams due to the smaller number of words in most comments. This is now included in line 116-117.

We did not stem or lemmatise tokens, again due to the small number of words in each comment, however we agree that lemmatisation is a good idea for future investigations. We considered that the use of the sentiment lexicons would cope with the common forms of the words, as stated in line 117-118.

In regards to the penetration of search terms, we feel confident we captured the majority of the videos that feature this description. We have added a statement within the paper (line 88-89) as suggested.

Thank you for your insightful suggestion regarding the reflection of the dual usage of 'like'. 1-gram tokens were used and as such we found this comment helpful and have added some discussion around YouTube culture to the paper (see line 281-286). We are hoping this provides the missing link between the YouTube algorithm and user engagement.

Data and code files should now be uploaded with the revised paper as a single pdf.

Reviewer 3
1. Thank you for your insight into the social science aspect of the use of ‘perception’ vs ‘opinion’. In this instance, we believe that the use of either term is effectively equivalent for the purposes of this study.

2. We agree that further statistical analysis would be helpful in interpreting the results, however as this was designed as a descriptive observational study we have decided not to go into this detail as it was beyond the scope of the study. As such, the data collected was not associated with a hypothesis but was collected to evaluate whether there was a potential connection between exotic animal content and perception. We have further studies planned where data will be collected based on hypotheses.

2.1 Thank you for your suggestion. The reviewer raises a valid point. In future, if we were to perform further statistical analysis, we would take this into account. However, at this stage the study was designed as a descriptive observational study. The aim was to analysis the relationship between sentiment (as an indication of public perception) and species conservation status and explore the association with the exotic pet trade. We wanted to avoid selection bias within the study. Being a descriptive study, we wanted to observe this potential relationship to suggest that it may be an area which could warrant further investigation. At this stage, the addition of the suggested model is outside the scope of this paper.

2.2 We believe this comparison would be interesting, however the aim of the study was to evaluate exotic animals in ‘free handling’ situations, wherein they featured some sort of interaction with humans. Therefore, the way we view it, the interaction with humans was more important than the setting, where we were aiming to imply that the setting was not necessarily as important in how these animals were viewed in this context (i.e. animals seen in captivity could still be considered desirable and promote demand for these animals as pets). We agree that this was not clearly explained within the discussion and therefore have modified it to account for this (line 311-315).

3. Thank you for highlighting the ambiguity associated with this point. We have added some additional description to explain why emoji analysis was important within this research (see line 288-296). Firstly, we argue that it can be difficult to gauge emotion from words alone, where emojis act as metadata. Additionally, emojis represent a global language, with the aim of avoiding cultural bias and misinterpretations of words detected in non-English languages.

<u>Introduction</u>
1. We agree with the reviewer to make the link between social media and the exotic pet trade stronger. We have revised the flow of the introduction, whilst ensuring the main points (as pointed out by the reviewer) have been explicitly addressed.

2. We agree with the suggestion to make the link between YouTube and the popularity of animal videos and have added a comment to address this.

3. In accordance with your comment, we have included further information as to why sentiment analysis techniques were chosen and why it was relevant to the study (see lines 64-71).

Line 32: The text has been edited as per the suggestion.

Line 32-34: These changes have been applied.

Line 32-39: A statement has been added to summarise this paragraph.

Line 40-41: We have made changes to the flow of the paragraph, as requested.

Line 42: This line has been edited to avoid confusion.

Line 40-47: A closing point has been added to this paragraph to help strengthen the link between the exotic pet trade and social media.

Line 51: We have changed ‘insufficiently regulated’ to ‘unregulated’ to avoid confusion.

Line 48-60: A new topic sentence which better addresses the main points of the paragraph has been included.

Line 61-64: We have altered the layout of the aim accordingly.

Line 64-66: Upon review, we agree that this statement was misplaced. We have moved it into a more appropriate position within the aim (line 35-36).

Methods

1. Further information regarding why the specific lexicons were chosen has now been included within the methodology (see line 131-140 and line 155-158). After exploring various dictionaries, we found that these lexicons provided the most appropriate information for our study.

2. Analysis was predominately based on evaluating sentiment trends generated and depicted through *gglot2*. In accordance with this, we firstly noted no statistical difference between the IUCN Red List Classifications in regards to sentiment (as shown in figure 2a and 2b) which suggested no clear distinction between the categories (interpreted as lack of public awareness of the significance of conservation status). In regards to changes in sentiment trends over time, we observed that sentiment remained consistently positive throughout time and was significantly different from zero based on the application of 95% confidence intervals (except for primate text sentiment in 2015, figure 3b).

Line 90: This has now been rewritten.

Line 91: You are correct in that tuber::get_comments( ) typically only extracts 100 comments at a time, however we designed code to bypass this, enabling the function to effectively be repeated to extract all comments. A comment has been included within the paper to address this (see line 107-109).

Line 109-111: Clarification regarding the sentiment parameters and lexicon selection has been included.

Line 120 – 121: The emoji sentiment lexicon published by Novak et al. was chosen as it provided suitable code to complement the requirements of the study and was novel work not detailed

elsewhere when the analysis was being carried out. The code itself was not altered but was merely written into R. A statement has been included in the methodology to describe this.

Results

1. As stated previously, although it would have been interesting to analyse the different response to captive animals compared to pets, the most important point is that regardless of the setting the animals were depicted in the interaction with humans ('free handling'). The idea behind this was that we wanted to suggest that despite the living situation of the animal (whether it was in a zoo or as a pet), public perception was similar and both contributed to increased demand for these animals. This was perhaps not very clear; hence, some revisions have been made to make this point stronger.

2. A table has been added to the supporting information regarding the number of comments extracted. If a user made multiple comments within the comments section, these were all included (as all comments were extracted). This includes both primary comments (main comments made) and secondary comments (comments made underneath primary comments). If the creator commented within the comments section, these were also included. A description has been included within the methods (line 109-111).

Table 1: The table legend has been updated to include further information, as requested.

Line 155-160: This information has been moved to the methods section.

Line 192 – 194: Thank you for your suggestion. However, upon analysing the boxplots, the results were not statistically significant from zero (with all plots intersecting zero), therefore the standard deviation was not included. The analysis was predominately based on the positive distribution of data. One of the main findings from the box plots was also surrounding how the classification groups overlap, suggesting the public either did not recognise the conservation classification associated with the species shown or did not care. 95% confidence intervals were applied and have been included within the supplementary code file.

Line 186: Thank you for highlighting this uncertainty. The methods section has been updated to make the sampling method clearer. A simple random sample was taken from each wild cat species using the set.seen(n) function in R to produce the 10,000 comments. Sub-sampling involved ensuring these comments were refined to only those containing at least one emoji (n>0) to provide a more meaningful analysis.

Line 186-189: This section has been revised.

Line 192-193: This section has also been revised.

Discussion

1. The additional headers within the discussion have been removed to help improve flow, as recommended. Further editing has been undertaken to try and make the aims clearer to the reader.

2. Thank you for highlighting this. We have aimed to re-emphasise the data and findings within the discussion to address this.

Line 225: As now stated within the discussion, ‘YouTube® comments serve as a gauge of public perception due to its wide-reaching accessibility and popularity, where several studies have demonstrated the impact social media platforms have on public opinion’ (line 271-273). Therefore, we believe that for the purposes of this study the comments extracted represented public opinion surrounding the depiction of exotic animals in YouTube videos.

Line 225-230: The beginning of the discussion has been revised to ensure better flow.

Line 233-234: Some of this information has been moved to the methods section as requested, whereas the interpretation remains within the discussion.

233-241: Thank you for your comment. We have aimed to incorporate the limitations of the sentiment software with the aims of our study, as suggested. In regards to the identification of sarcasm, it was difficult to derive a subset of comments which demonstrated this given the nature of our data, however studies have been referenced regarding which emojis are commonly used in the expression of sarcasm as a generalised example (line 293-295).

Line 304-331: Thank you for your insight regarding the recommendations made, we were unaware of projects such as Wildbrook and after some research have decided to include it within the discussion. Additional simpler changes have also been suggested, however there are likely other approaches which could be considered also.

Figures

Changes have been made to the figures, as suggested.
