# Supplementary material for: Is YouTube promoting the exotic pet trade? Analysis of the global public perception of popular YouTube videos featuring threatened exotic animals
Source: PLoS One. 2021 Apr 13;16(4):e0235451. doi: 10.1371/journal.pone.0235451 (PMC8043400; doi:10.1371/journal.pone.0235451)
Supplement: Attachment — Submitted filename: Review comments round 3.docx [file pone.0235451.s008.docx]

Dear Reviewer,

Thank you very much for your valuable comments, we have substantially modified the manuscript following your suggestions and hope this improved version will meet your expectation.

Best regards,

ALC

The reviewer’ comments are addressed in red below.

Reviewer #4: I think the topic of investigation is of broad interest and it would be important to publish these results. That said, more work is necessary, especially in the presentation of the results. Some of the issue raised by previous reviewers are still not fully addressed (statistical analysis, hypotheses), and I provide more suggestions.

In order to address these comments we have included mixed models to assess which variables were associated with text and emoji sentiments

- Lines 72-76. I understand here the critique of the reviewers. Even if you want to have a descriptive paper, you inevitably test hypotheses since you expect a difference in perception of exotic pets based on their IUCN status, and you also expect a trend with time. You can justify why in here.

We have included more analyses in the paper and have reworded objectives accordingly.

- Lines 189-190. Table S2 does not really show a right-skewed distribution. There is no index of skewness reported. The median value is much lower than the mean, so the majority of videos have lower comments than the mean (so probably left-skewed), and there seems to be outliers on the right. But I would expect a histogram or a skewness index if you talk about skewness.

This is correct, we have hence reworded this

- Table 1. For some genera, you probably have more than one species (it is not clear if you did not identify them). It should be clear how you handled with this frequent situation as for some genera (e.g. Macaca) you can have IUCN classifications ranging from Least Concern to Critically Endangered. So having Macaca spp. and the relative IUCN Red List category Endangered is probably incorrect unless you considered only Endangered species. That is true for other genera as well as you do not present the species. In the table, there is also a typo (Varecia variegata not variegate). If you have the genus you should also add spp. if you consider more species of the same genus (and of course they should all be with the same IUCN status).

We have clarified the IUCN classifications of this group and included this in the paper (and corrected the spelling mistake; thank you for noticing).But, you are correct, there were several Macaca species, for this situation, IUCN listing was based on most severe status of identified species

- Lines 210-211. I do not understand this result. You selected the videos with wild cats and primates, so not sure what are these results relative to dogs. Do you mean that wild cats and primates had interactions with dogs in 17.9% of the videos? Can we know more about which species?

The interpretation is correct, this has now been reworded – line 220 now

- Line 216. Likewise is not appropriate here as IUCN Red List and CITES are not going on the same direction.

This has been reworded to correct (line 225)

- Text sentiment analysis section. I would expect a word frequency analysis, that can be interesting and quick to add, and can show more about the content of the comments.

To address this we have included the mixed models for text and emoji sentiment

- Lines 229-231. This statement would require a statistical analysis. You also need to consider the other issue relative to IUCN Red List status, comment to table 1.

We have reworded this and changed the figure – now starting line 245

- Lines 246-247. Here you need statistical analyses to confirm your statements.

This has been reworded (line 276 start)

- Line 249. It seems neutral as it is close to 0 (unless it is statistically significant from 0).

This has been deleted and results presented based on the mixed models

- Line 270-271. Here you report the same phrase as in the results but you do not explain why it is important, you do not discuss this result, you just repeat it.

Added interpretation - Now line 305

- Lines 279-280. I do not see this from your results.

These results are included in line 230

- Line 299. You do not really discuss the implications of your results. You found that the perceptions were positive, but what does it mean? What does it entail? You should move here the paragraph that is now almost at the end of the discussion

We have moved the paragraph as suggested (line 345) and combined with discussion as per mixed method models

- Line 301. You did not really test this

This is true, we have now deleted this statement

- Line 306. Did you recognize the species? As there are so many macaques with so many different statuses.

The results are based on the identified species (as per mentioned above)

- Lines 359-374. I think this paragraph should go above as this is your main discussion on your findings.

Moved as suggested - line 344

- Line 369. But this is only relative to slow lorises, how can this impact your analysis as you have many species and only 7 videos of pygmy slow lorises.

This	was probably not clear from the results included. We suggested this due to the results in the new and more detailed figures included now. This includes figure 2 (slow loris the only species with negative sentiment) and figure 5 (reduction in primate sentiment in 2015).

- Line 420. IUCN Red List status ranged from Least Concern to Critically Endangered, so not all the species you considered are threatened. You should rephrase it.

This comment is correct; however, the results show that all groups on the IUCN red list have a positive median sentiment. We have tried to clarify this.

- Figure 2a. The IUCN categories should be upper case. In the x axis label you say Mean Sentiment Score but you show box plots (i.e. median, quartiles, range, and outliers). I suggest changing the figures to have a better presentation, maybe use theme(bw), can try different themes in ggplot2.

Figure has been changed (now fig 3)
