# Supplementary material for: Is YouTube promoting the exotic pet trade? Analysis of the global public perception of popular YouTube videos featuring threatened exotic animals
Source: PLoS One. 2021 Apr 13;16(4):e0235451. doi: 10.1371/journal.pone.0235451 (PMC8043400; doi:10.1371/journal.pone.0235451)
Supplement: Attachment — Submitted filename: Response to Reviewers.docx [file pone.0235451.s009.docx]

**Response to Reviewer 4**

Abstract: The abstract has been altered to incorporate the new findings.

Line 113: This was corrected.

Line 116. The terminology was checked accordingly and we believe it to be correct now.

Line 175-179: The command used to run the mixed model has now been added to the methods section (line 181-183). We used the lmer function which is a general linear mixed model. Furthermore, we understand that repeat measures and random intercepts are not the same, but we found no evidence for the need of a temporal effect. 

Line 190: Thank you for pointing this out, it has been corrected.

Results: Thank you for your suggestion, the individual sections have been reduced to integrate the results better.

Line 253. Thankyou for noticing this, it has been corrected.

Line 312: This has also been corrected.

Line 321. This has been rephrased with more certainty.

Lines 343-347. This paragraph has now been integrated into the results section where it appears to be more suitable.

Lines 324, 344: Data can be obtained from the authors as required upon request, hence was not included within the supplementary information.

Lines 430-432: The higher median text sentiment in ‘Least Concern’ species was mentioned earlier in the text (line 333-335), wherein the conclusion has now been reworded to make this clearer to the reader.

Figure legends: Thank you for pointing this out, ‘quantiles’ has been changed to ‘quartiles’ throughout the manuscript.

Thank you for your time and suggestions to help improve our manuscript.
